# Parametric Study of an Electroosmotic Micromixer with Heterogeneous Charged Surface Patches

**DOI:** 10.3390/mi8070199

**Published:** 2017-06-23

**Authors:** Faheem Ahmed, Kwang-Yong Kim

**Affiliations:** Department of Mechanical Engineering, Inha University, Incheon 22212, Korea; faheeem@live.com

**Keywords:** micromixer, electrokinetics, electroosmotic flow, charged surface patches

## Abstract

A T-shaped micromixer featuring electroosmotic flow with heterogeneous charged surface patches on the channel walls was analyzed, and an improved design was proposed to enhance mixing performance. Numerical analysis was performed using steady Navier-Stokes equations with an additional electrokinetic body force. The numerical results for species concentration were validated with available experimental data. A parametric analysis of the micromixer was performed by varying channel height, channel width, patch width, and externally applied voltage. The effects of these parameters on the flow structure and mixing performance were analyzed in detail. A quantitative measurement based upon the mass variance was employed to quantify the mixing performance. Numerical results of the parametric study were used to propose an improved micromixer design with spacing between adjacent charged patches. The proposed design provided a more favorable flow structure to allow for enhanced mixing performance.

## 1. Introduction

In recent years, noticeable advancements in microfluidics have been achieved in application to micro total analysis systems (μTAS) and lab-on-a-chip devices [1]. When compared to their macroscale counterparts, these devices offer significant advantages like lower cost, higher performance, and reduced sample consumption. Thus, these microsystems gained considerable popularity and increased interest among biologists, chemists, and engineers, both in academia and industry. Device portability, enhanced mixing, and shorter analysis time are critical criteria of any efficient μTAS. Therefore, a rapid and efficient mixing process plays a key role in the development of microdevices. However, since characteristic dimensions of microfluidic systems are typically in μm scale, flow regime in such small dimensions is characterized by low Reynolds and Peclet numbers, and thus the mixing primarily relies on the slow process of molecular diffusion rather than advection. This requires larger channel length and mixing time to achieve homogenization of the species. Therefore, enhancing the efficiency of the mixing process is the primary objective in designing micromixers for μTAS applications to ensure the reduction in analysis time and size of the devices.

The micromixers can be categorized into active and passive mixers depending upon their mixing mechanism [1]. Passive micromixers make use of geometry to produce complex flow fields for efficient mixing. They do not require any moving parts and can be easily manufactured in both planar and three-dimensional configurations. Passive micromixers rely on the mechanisms like split and recombination of flow, flow separation, chaotic advection and hydrodynamic focusing. Generally, passive mixers are preferred because of their simplicity of design, ease of manufacturability and system integration. However, passive mixing methods present less efficient mixing than active type mixers, and thus have the disadvantages of longer mixing time and channel lengths, and high pumping power requirements. Active micromixers use external energy or stimuli to enhance the mixing of fluid species. They feature either moving parts [1] or externally applied forcing functions such as electric fields [2], magnetic fields [3], and pressure [4]. Though they require relatively shorter mixing time and channel length, active micromixers are more complicated and difficult to integrate compared to the passive ones, especially if they require any moving part. However, active micromixers featuring simple electroosmotic flow have a simpler design without any moving parts, and thus they offer enhanced flow control with ease of manufacturing and implementation.

A lot of research has been carried out on electroosmotic micromixers. Different strategies were suggested and tested to manipulate and control the fluid motion in these micromixers; Chen and Cho [5] introduced the concept of an embedded non-conducting obstacle on the microchannel wall. Heterogeneously charged obstacles were used by Chang and Yang [6] to improve the mixing performance. Slanted grooves were used by Johnson [7]. The mixing mechanism using heterogeneous surface charge patches is an effective and relatively easy to be implemented for mixing enhancement. The heterogeneous surface charge could be introduced on the sidewall and/or the bottom of the microchannel [8,9,10]. This technique of introducing surface heterogeneity capitalizes on induced localized non-axial flow structure or vortices to achieve high mixing efficiency. Similarly, localized flow vortices could also be generated by using non-uniform surface potential/charge. Previous works [9,10] revealed how these induced non-axial flow structures could be manipulated by introducing heterogeneity at the bottom of the microchannel to achieve high mixing performance. Biddiss et al. [8] compared the mixing performances of different configurations of heterogeneous charge patterns and determined that a staggered pattern is optimal for a very low aspect ratio geometry. Using particle tracking method, Chang and Yang [9] investigated mixing performances of three patterns: diagonal, asymmetric herringbone and symmetric herringbone.

In the present work, a configuration of staggered heterogeneous patches in an electroosmotic micromixer was investigated numerically to develop a better understanding of the mixing mechanism inside the micromixer and finally to enhance the performance of the micromixer. This pattern of patches was used in a previous work [8], where an in-depth analysis of flow structure and impact of surface heterogeneities on mixing performance was not performed. Performance of the micromixer was evaluated in a parametric study by solving three-dimensional Poisson-Boltzman and Navier-Stokes equations for a pure electroosmotic flow with a diffusion-convection model for species concentration. Effects of electrical double layer (EDL) and applied electric field on the flow were incorporated by introducing corresponding body force into Navier-Stoke equations. Findings of this study were utilized to develop a micromixer design which can provide enhanced mixing performance over shorter channel length.

## 2. Micromixer Model

**Figure 1:**
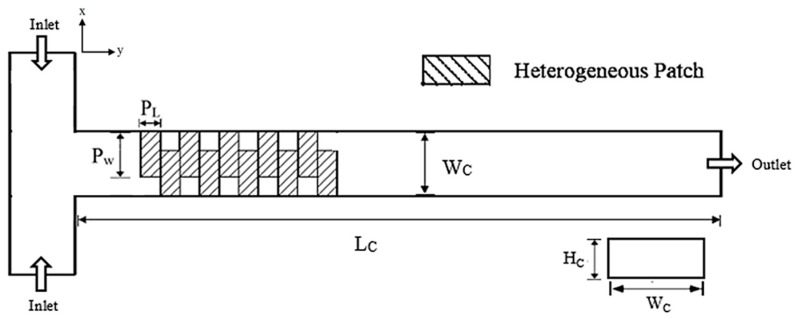
Schematic diagram and geometric parameters.

The two-dimensional schematic of the micromixer investigated in the present study is shown in Figure 1. The main channel, which consists of a rectangular cross section with a low height-to-width ratio, extends along the axial length. Dimensions of the microchannel are described in Table 1. The length of the microchannel was set as 2400 μm. The channel surface is assumed to have a uniform zeta potential of −42 mV, except for positively charged heterogeneous patches, which spread along the axial length on the channel base. These patches were placed along the axial length as shown in Figure 1. In this study, the number of heterogeneous patches was fixed at 10. The zeta potential for these heterogeneously charged patches was set as +42 mV. The top layer of the channel was set to be passivated (zero surface charge).

**Figure 2:**
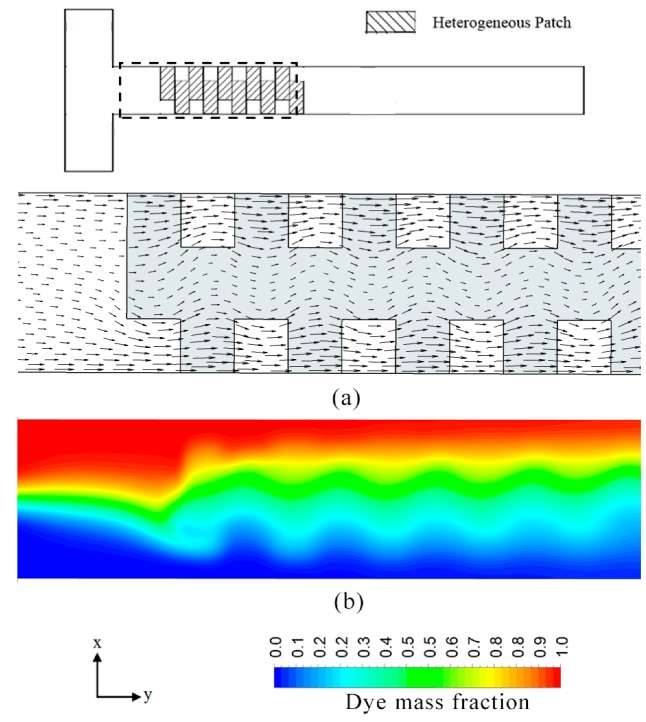
Mixing in the reference micromixer on *x*-*y* plane at *E* = 400 V/cm: (**a**) velocity vectors at *z*/*H*_c_ = 0.33 and (**b**) dye mass fraction distribution at *z*/*H*_c_ = 0.5.

Under the application of the external electric field, a homogenous surface with negative zeta potential drives the flow along the axial direction of the microchannel. On the other hand, the heterogeneous surface with positive zeta potential tends to force the fluid into the opposite direction. The addition of heterogenous patches over a homogenous surface tends to induce transverse flow components in the stream, resulting in localized rotational flows thus introducing an advective component to diffusion-dominated mixing. The flow pattern for the reference case geometry is described in Figure 2.

## 3. Electrical Double Layer and Numerical Model

**Figure 3:**
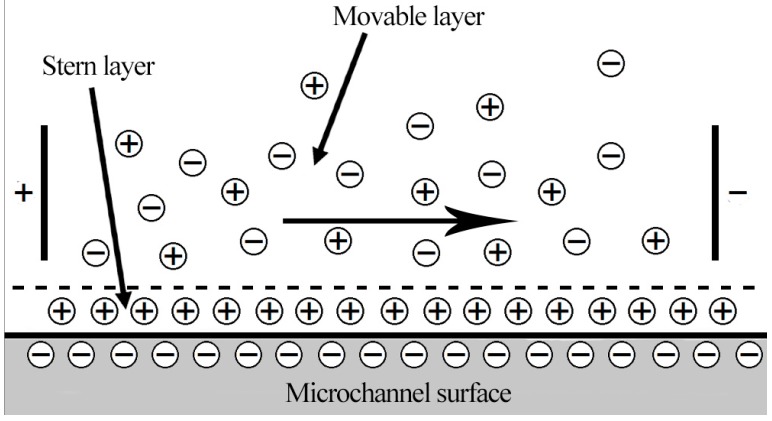
Schematic of electric double layer.

Fluid bulk motion induced by externally applied electric field in the presence of EDL near the surface of the microchannel, was modeled as a pure electroosmotic flow. The walls of the microchannel in most μTAS systems are made of dielectric material (e.g., silicon). When an aqueous solution comes in contact with the channel wall, the interaction yields net surface charge density. The surface charge density depends on the surface hydrolysis, adsorption or dissolution of ions and other reactions. The oppositely charged ions (counter ions) in the aqueous solution move towards the wall to form an EDL, as shown in Figure 3. The EDL has counterions in excess, while outside EDL both coions and counterions have uniform concentration. If an external electric potential is applied across the microchannel, a moveable layer of EDL gets activated, and movement of ions takes place. In the case of steady state, a plug-like fluid flow is generated as a result of the balance between the electroosmotic force on the ions in the diffuse layer and viscous drag force.

Mathematically, the electroosmotic force can be treated as a body force and introduced in the Navier-Stokes formulation [11,12]. Hence, the fluid flow is modeled using the continuity equation and the Navier-Stokes equations with an additional body force term due to the applied electric field:

Mass conservation equation:

(1) $$\nabla \cdot \left(\rho \vec{V}\right)=0$$

Navier-Stokes equations:

(2) $$\rho_{f}\;\left(\vec{V}\cdot \nabla \right)\vec{V}=\;-\nabla p+{\;\mu }_{f}\;\nabla^{2}\vec{V}+\;\rho_{e}\;E$$

The net charge density $\rho_{e}$ can be described by the distribution of charges in the EDL. Three-dimensional extension of the EDL model suggested by Arulanandam and Li [12] was used to describe the charge density distribution as:

(3) $$\nabla^{2}\psi =-\;\rho_{e}/\varepsilon$$

Electrical potential and ion distribution are not significantly affected by convection in low Reynolds number (Re < 10) flows [13]. The Reynolds number (Re=ρfud/μf) is calculated using average velocity at channel exit (*u*) and hydraulic diameter of the channel cross section (*d*). Hence, electrical potential and ion concentration can be described using the equilibrium Boltzmann distribution equation [14];

(4) $$n_{i}=n_{\infty }\;\exp\left(-\frac{z_{b}e}{k_{b}T}\psi \right)$$

In case of symmetric electrolyte, the net charge density can be described as:

(5) $$\rho_{e}=-\;2n_{\infty }\;z_{b}e\;\sin h\left(\frac{z_{b}e}{k_{b}T}\psi \right)$$

Substituting Equation (5) into Equation (3), Poisson-Boltzmann equation can be obtained as:

(6) $$\nabla^{2}\psi =\frac{2n_{\infty }\;z_{b}e}{\varepsilon }\;\sin h\left(\frac{z_{b}e}{k_{b}T}\psi \right)$$

The permittivity of the aqueous solution was taken as ε = 80. Since the externally applied electric field in this study does not exceed 1000 V/cm, the Joule heating effect is negligible and thus was ignored [15]. In this work, 10^−3^ M KCl electrolyte solution was used as the working fluid. The physical properties of the working fluid were set as follows; ρ*_f_* = 10^3^ kg/m^3^ and μ*_f_* = 10^−^^3^ Ns/m^2^. The diffusivity coefficient of the scalar dye was set as 3 × 10^−^^10^ m^2^/s.

The flow was assumed to be laminar and steady, and the mixing process was assumed to be isothermal. No-slip boundary conditions were applied at interior walls of the channel. As the flow was modelled totally electrokinetically driven, the pressures at the inlets and outlet were specified as zero. Electric field **E** was applied uniformly across the entire channel length.

The numerical simulations were carried out using a commercial finite volume solver CFX 15.0 [16] with added customization to incorporate the electric potential equations. The solver used the coupled algebraic multigrid method [17]. For convergence criteria, the root-mean-square residuals for all governing equations were set to 1.0 × 10^−6^. The computations were performed by the Intel Core I7 CPU having 3.6 GHz CPU, and the computing time per single simulation was about 8–48 h with approximately 600–1600 iterations for convergence varying in accordance with the preset condition.

## 4. Numerical Model Validation

**Figure 4:**
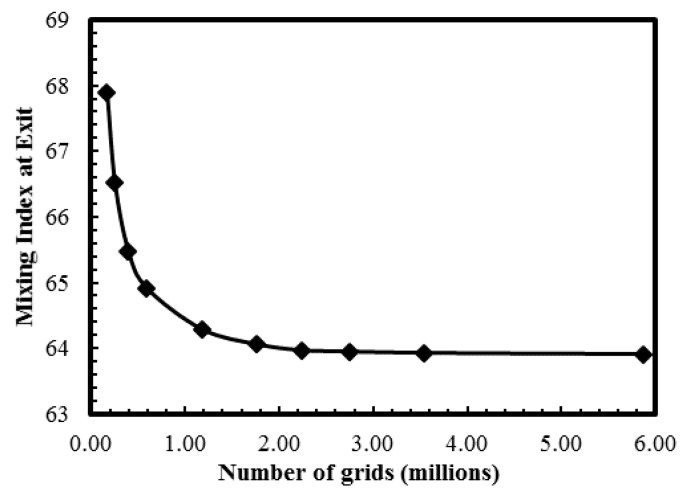
Grid-dependency test for the mixing index at the exit.

An unstructured tetrahedral grid system was used in this study. The grid structure was set up in a manner that would ensure full capture of the flow complexity induced in the electroosmotic fluid flow. In order to ensure the grid independence of the numerical solution, an extensive grid-dependency test was performed to determine the optimal number of grids. Ten different grid systems with number of grids ranging from 3.95 × 10^5^ to 5.86 × 10^6^ were tested for the reference geometry, and the optimal number of grids was determined to be 1.76 × 10^6^. The results of the grid-dependence test are shown in Figure 4. At the channel exit, negligible variation in the mixing index (0.14%) was observed between the optimal grid and the next finer grid with 2.23 × 10^6^ nodes.

**Figure 5:**
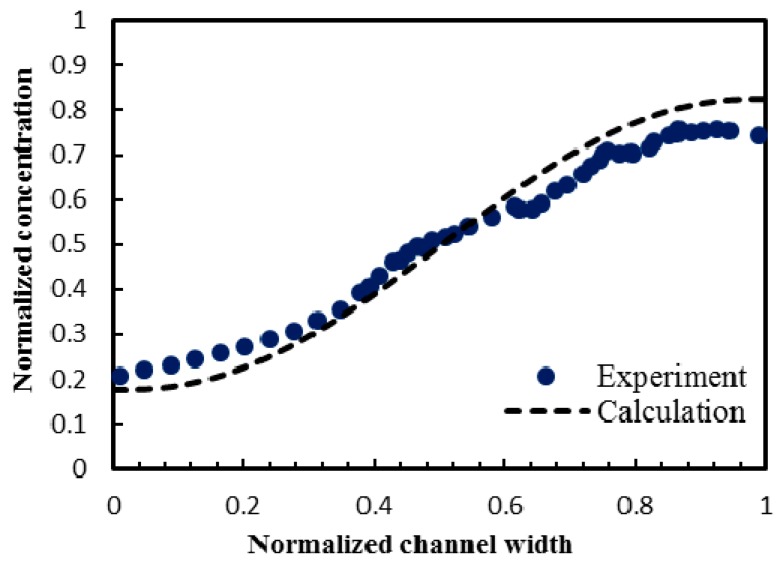
Validation of the numerical results for normalized concentration profile across the channel width using experimental data (Wu and Li [18]).

The numerical model used in this work for the analysis of the electroosmotic flow was validated using experimental data obtained by Wu and Li [18] for electroosmotic flow in a microchannel. The electroosmotic model was tested against the homogeneous microchannel with non-conducting symmetric hurdles, as presented in the reported work. The channel had a width of 300 μm and a height of 36 μm. Values of the diffusion coefficient and the externally applied electric field were 4.37 × 10^−10^ m^2^/s and 50 V/cm, respectively. The results from the present numerical model for species concentration profile across the channel width near channel exit (at axial length = 2000 μm) were in good agreement with the experimental data that is shown in Figure 5. Average relative error of 0.59% was observed between the two data sets.

## 5. Performance and Geometric Parameters

The variance of the mass fraction of dye in the main channel was calculated to evaluate and analyze the mixing performance. The variance is based on the intensity of segregation, using the mean concentration. Mixing index (MI) based on the mass variance method was used to measure the mixing performance. The variance of mass fraction of the mixture at a cross-sectional plane normal to the flow can be mathematically described as:

(7) $$\sigma =\sqrt{\frac{1}{N}\overset{N}{\underset{i=0}{\sum }}{\left(c_{i}-{\bar{c}}_{m}\right)}^{2}}$$

where *N* represents the number of sampling points, $c_{i}$ is mass fraction at sampling points *i*, and $c_{m}$ is the optimal mass fraction. Mixing index based on the mass variance is defined as:

(8) $$\mathrm{MI}=\left\{1-\sqrt{\frac{\sigma^{2}}{\sigma_{\max}^{2}}}\right\}\times 100$$

Here, σ stands for the standard variation of the species concentration across the channel in a cross section at any specified location, and $\sigma_{\max}$ is the maximum standard variation. The higher mixing index indicates better mixing. Therefore, mixing index would assume a value of zero for a completely separate stream and unity for a completely mixed stream.

The geometric parameters indicated in Figure 1 were tested in the parametric analysis; ratio of channel width to channel height (*W*_c_/*H*_c_), ratio of patch length to channel width (*P*_L_/*W*_c_), and ratio of patch length to channel width (*P*_w_/*W*_c_). Additionally, ratio of externally applied voltage to zeta potential (*V*/ζ) was also tested as an operation parameter. The ranges of these dimensionless parameters studied in the parametric analysis are described in Table 1.

## 6. Results and Discussion

**Figure 6:**
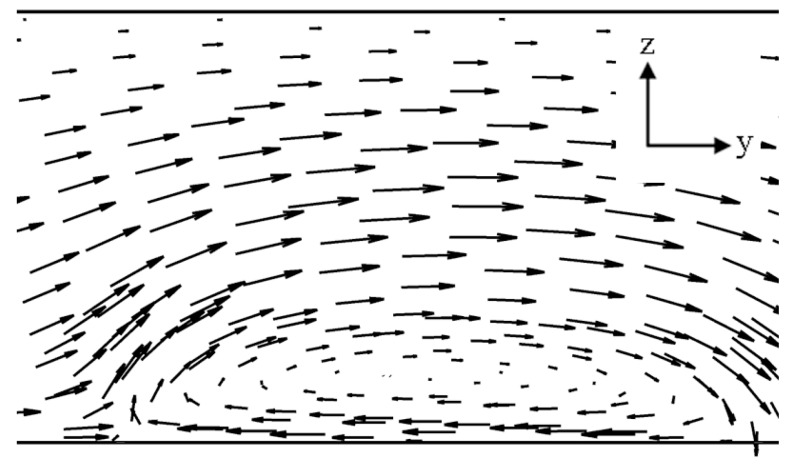
Vector plot at a *y*-*z* plane over a single heterogeneous patch.

**Figure 7:**
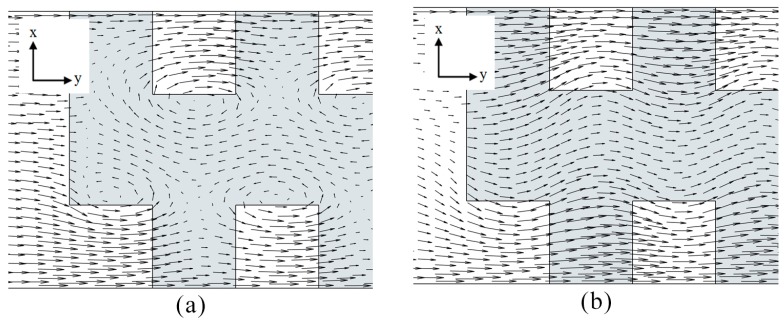
Velocity vectors on *x*-*y* plane: (**a**) *z*/*H*_c_ = 0.2 and (**b**) *z*/*H*_c_ = 0.45.

In this study, impact of the selected four parameters on the flow structure and mixing performance of the electroosmotic microchannel, featuring heterogeneous staggered patches, was analyzed. The multiple staggered heterogeneous patches inside the homogeneously charged channel cause flow vortices on *y*-*z* and *x*-*y* planes. Each heterogenous patch induces a vortex on the *y*-*z* plane as shown in Figure 6, and another vortex on the *x*-*y* plane at its lateral edge shown in Figure 7a. The later vortex generated by the alternately placed patches plays a critical role in enhancement of mixing performance by enlarging interferential area between the two mixing fluids.

Since heterogenous patches were introduced at the bottom of the microchannel, strength of the vortices reduces as the channel height increases. Figure 7 shows the velocity vector plots on the *x*-*y* planes at two different *z*/*H*_c_. These plots indicate that the induced flow vortices decay with the increase in the channel height, and finally change to a wavy motion as shown in Figure 7b. For *z*/*H*_c_ larger than 0.33, the flow vortices are critically weakened, and thus do not contribute significantly towards the mixing enhancement. Therefore, *z*/*H*_c_ = 0.33 can be considered as the optimal channel height for heterogeneously charged electroosmotic micromixer featuring a passivated top layer. It should be noted that the optimal channel height can be increased by introducing additional heterogeneous charged patches on the non-passivated top layer. These top layer patches would induce additional vortices, thereby yielding a higher mixing performance. The induced wave at *z*/*H*_c_ = 0.33 is shown in Figure 2. This wave can be characterized using three factors, namely, wave amplitude, wavelength and shape form. The profile of the induced wave plays a critical role in the mixing. This study includes a qualitative analysis on the effects of the selected parameters on the flow structure of the induced wave motion.

**Figure 8:**
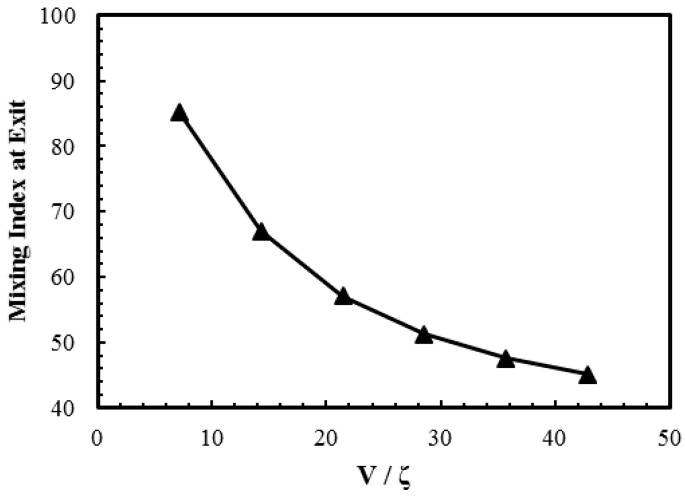
Effect of *V*/**ζ** on mixing index at the exit.

The main driving force for the electroosmotic flow is determined by the electric field applied across the channel. The electric field determines magnitude of the average flow velocity, and has a very strong influence on the induced flow vortex and mixing of fluid species; a low value of electric field tends to enhance the diffusive mixing by intensifying vortex and extending the residential time of the fluids in the channel. In order to study the effect of an externally applied electric field on the mixing performance, the reference design was tested over a wide range of externally applied voltages, as shown in Figure 8. This figure highlights the trend between the externally applied voltage normalized by zeta potential and the mixing index measured at the exit of the microchannel. Higher voltage causes a decrease in the residential time which decreases the mixing performance considerably, especially in the case of the inefficient heterogeneously charged pattern. It can be observed that, as the normalized voltage decreases to below 28.5, the mixing index increases rapidly. In contrast to the mixing index, Reynolds number increases linearly as the applied voltage increases. Normalized voltage (*V*/ζ) values of 7.14 and 42.8 yield Reynolds numbers of 0.01 and 0.04, respectively. The *V*/ζ value of 28.5 which corresponds to the electric field value of 400 V/cm, was selected as the reference value in the parametric study.

**Figure 9:**
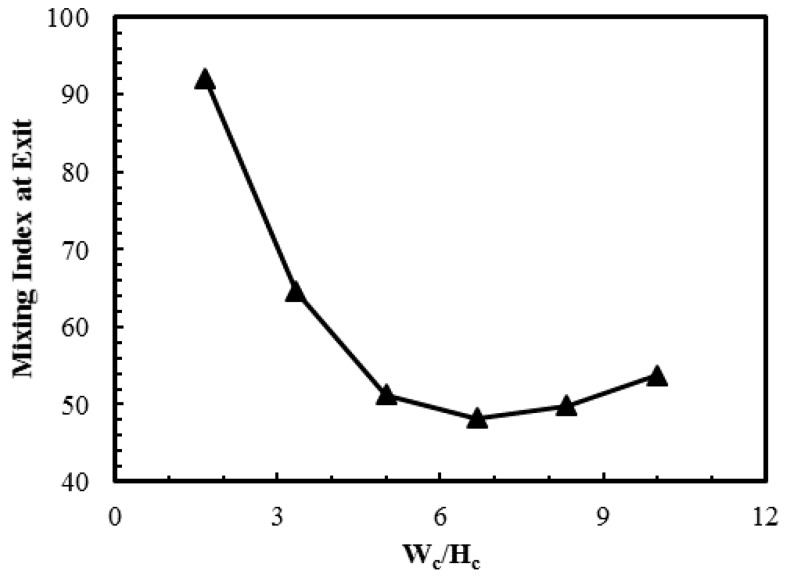
Effect of *W*_c_/*H*_c_ on mixing index at the exit.

**Figure 10:**
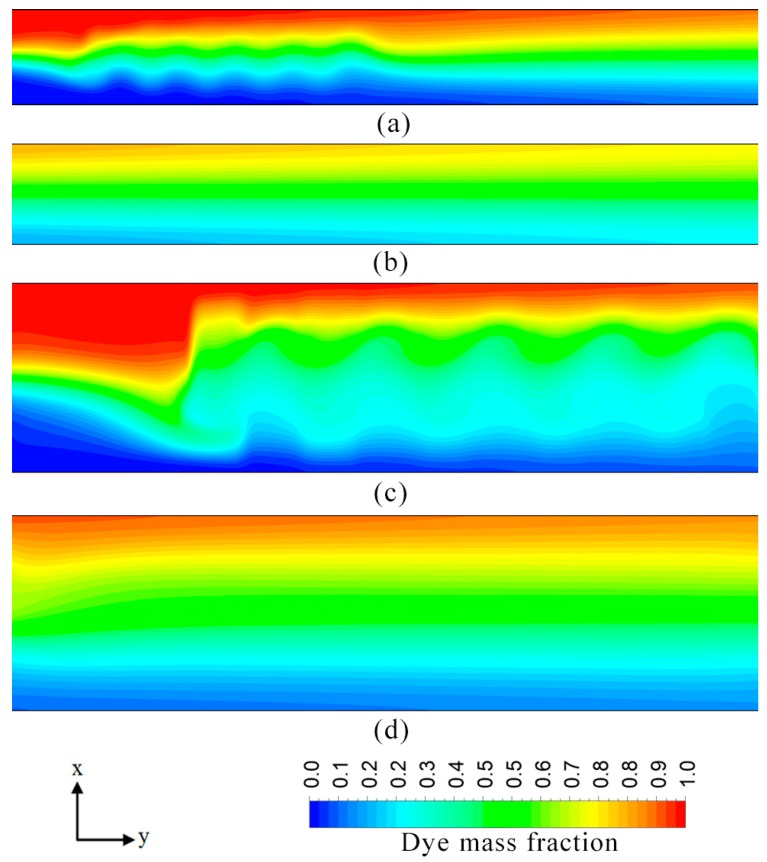
Dye mass fraction distributions on *x*-*y* plane at *z*/*H*_c_ = 0.5 for *E* = 400 V/cm: (**a**) *W*_c_/*H*_c_ = 3.33; near inlet (*x*/*L*_c_ = 0.05–0.42); (**b**) *W*_c_/*H*_c_ = 3.33; near exit (*x*/*L*_c_ = 0.56–1.0); (**c**) *W*_c_/*H*_c_ = 8.33; near inlet (*x*/*L*_c_ = 0.15–0.5); and (**d**) *W*_c_/*H*_c_ = 8.33; near exit (*x*/*L*_c_ = 0.6–1.0).

Effect of channel aspect ratio (*W*_c_/*H*_c_) on the performance was analyzed by varying the width of the channel. Figure 9 shows variation of mixing index at the outlet with the aspect ratio. The mixing performance of the microchannel decreases steadily as the channel aspect ratio increases from 1.65 to 6.65. However, further increment in the value of aspect ratio results in a small yet observable increase in the value of the mixing index. An increase in the channel width impacts the mixing performance negatively since it increases the distance between the opposite near-wall streams of the two fluids. Therefore, the mixing process which relies primarily on diffusion gets hindered excessively. Dye mass fraction distributions in the microchannel for two different aspect ratios are illustrated in Figure 10a–d. Increase in the channel aspect ratio increases width of the wave. However, the aspect ratio does not significantly affect the other features, such as amplitude, wavelength and waveform, and thus does not contribute excessively to mixing. The better mixing performance of *W*_c_/*H*_c_ = 3.33 compared to that of *W*_c_/*H*_c_ = 8.33 can be attributed to the smaller diffusion distance. The former case shows a comparatively lesser degree of homogenization of species in the mixing region, but higher a degree of mixing near the exit. The slight increase in the mixing index for the aspect ratio values higher than 6.65, can be explained by the fact that an increase in the channel width is accompanied by the increase in the total area of the heterogeneously charged surface at the channel base wall. This results in the relatively lower bulk velocity and slightly higher retention time, and thus the mixing performance is improved slightly.

**Figure 11:**
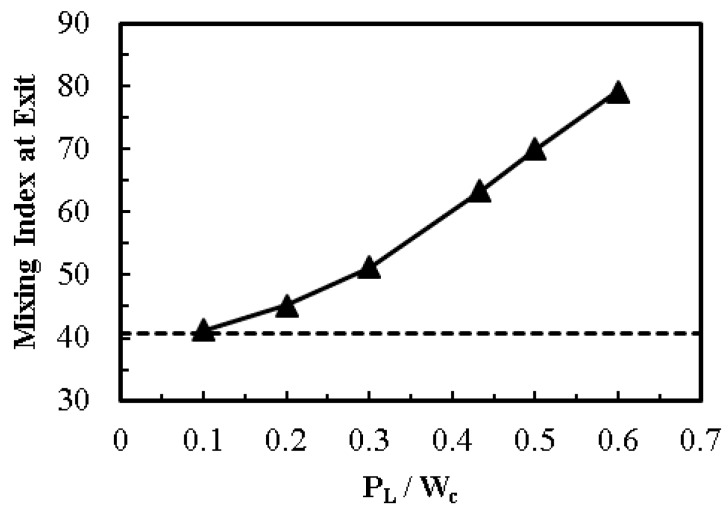
Effect of *P*_L_/*W*_c_ on mixing index at the exit (homogeneous channel, dashed line; heterogeneous channel, solid line with symbols).

**Figure 12:**
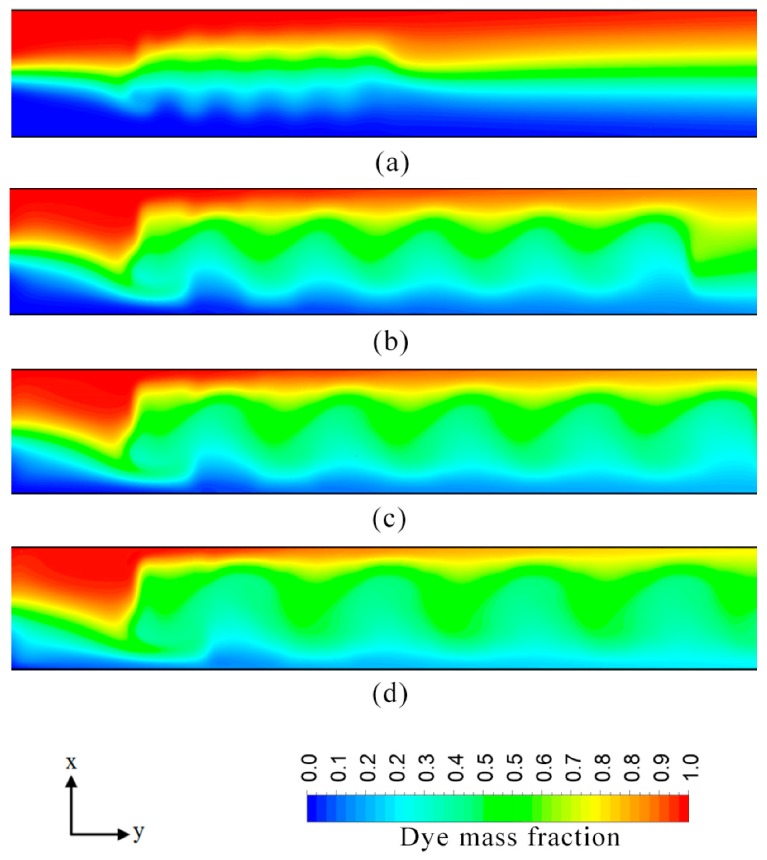
Dye mass fraction distributions on *x*-*y* plane along the channel length at *z*/*H*_c_ = 0.5 for *E* = 400 V/cm: (**a**) *P*_L_/*W*_c_ = 0.2; (**b**) *P*_L_/*W*_c_ = 0.43; (**c**) *P*_L_/*W*_c_ = 0.5; and (**d**) *P*_L_/*W*_c_ = 0.6.

The ratio of patch length to channel width (*P*_L_/*W*_c_) significantly affects the mixing performance of the electroosmotic microchannel, as shown in Figure 11. Increasing the patch length rapidly increases the mixing index. An increase in the value of *P*_L_/*W*_c_ from 0.3 to 0.6 increases the mixing index by 28%. Figure 12 shows dye mass fraction distributions in the microchannel for different *P*_L_/*W*_c_. Alteration in the value of patch length (*P*_L_/*W*_c_) significantly modifies the wave motion. An increase in the value of surface patch length (*P*_L_) increases the wavelength and amplitude of the induced wave resulting in the expansion of the interfacial area. The other noticeable effect is the reduction of distance between interfacial area and each near-wall stream of a fluid, which enhances diffusion and mixing performance. In this study, value of *P*_L_/*W*_c_ up to 0.6 was analyzed. This value can be extended further to intensify the homogenization process. The increased patch area would also slow down the bulk velocity inside the microchannel. Excessive heterogeneity (patch area) may result in a very low Reynolds number which may render the micromixer unsuitable for certain practical applications.

**Figure 13:**
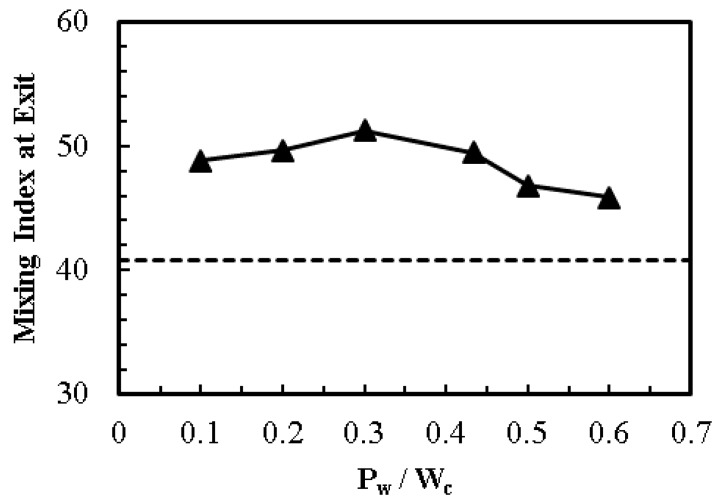
Effect of *P*_w_/*W*_c_ on mixing index at the exit (homogeneous channel, dashed line; heterogeneous channel, solid line with symbols).

**Figure 14:**
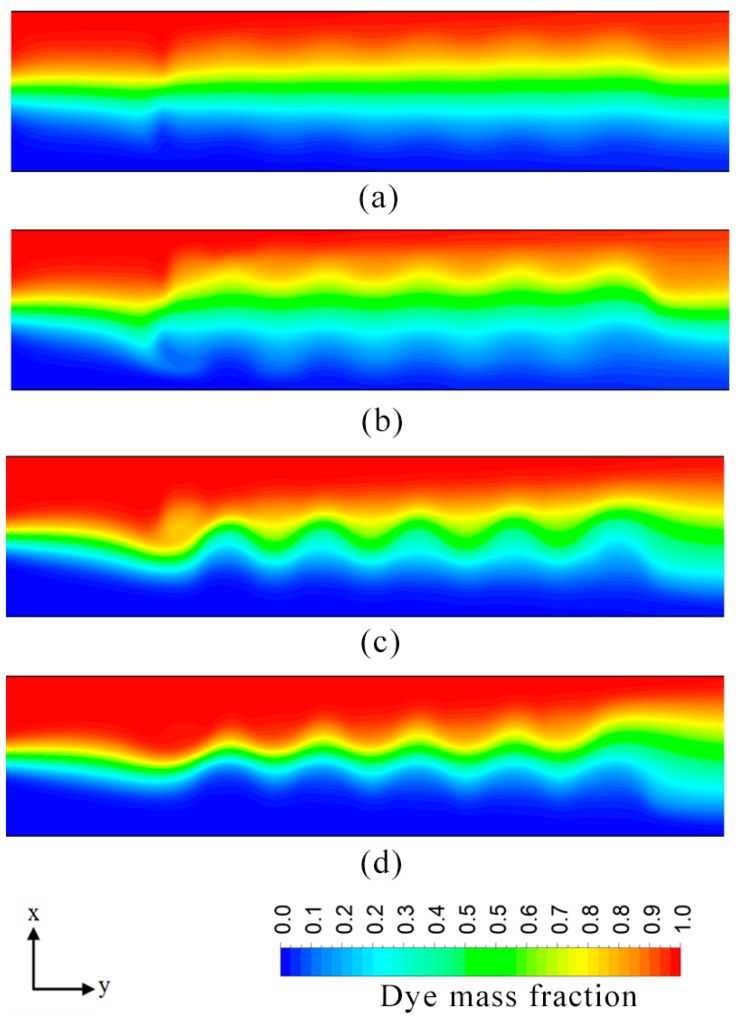
Dye mass fraction distributions on *x*-*y* plane along the channel length at *z*/*H*_c_ = 0.5 for *E* = 400 V/cm: (**a**) *P*_w_/*W*_c_ = 0.2; (**b**) *P*_w_/*W*_c_ = 0.43; (**c**) *P*_w_/*W*_c_ = 0.5; and (**d**) *P*_w_/*W*_c_ = 0.6.

Changing the ratio of patch length to channel width (*P*_w_/*W*_c_) does not show significant effect on mixing performance, as shown in Figure 13. The mixing performance is at its maximum at *P*_w_/*W*_c_ = 0.3. Deviation from this value causes a decline in the overall mixing performance of the micromixer. Dye mass fraction distributions for different values of *P*_w_/*W*_c_ are shown in Figure 14. It can be observed that excessive increase in the patch width decreases the width of the homogenous sections wedged between two adjacent heterogenous patches. These reduced homogenous sections are unable to induce the fluid velocity required to achieve strong flow vortex, as shown in Figure 7. Similarly, a decrease in the patch width below an optimal value brings the induced vortices closer to the centerline of the microchannel. Consequently, the reduced patch width increases the amplitude of induced wave, and thus increases the widths of fluid streams in the near-wall regions, resulting in a decrease in the mixing performance.

**Figure 15:**
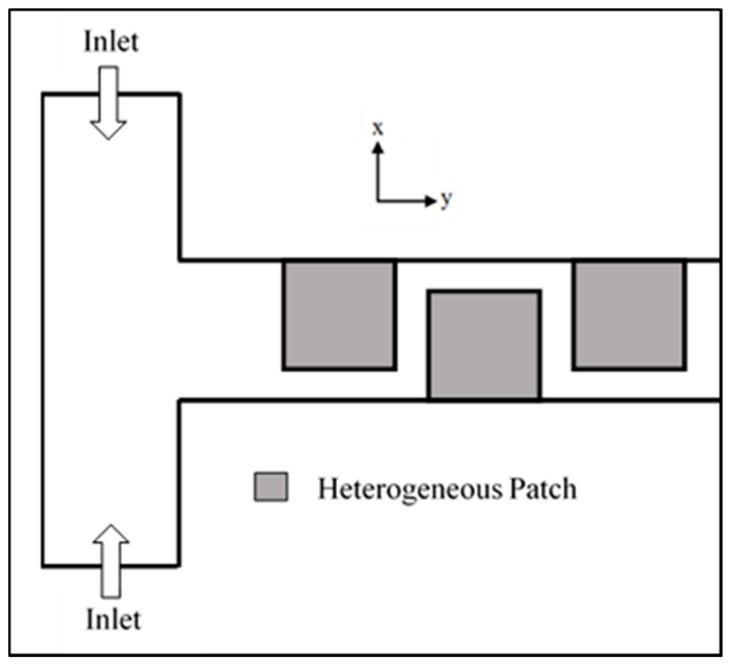
Schematic diagram of the new micromixer design with spacing between adjacent charged patches.

**Figure 16:**
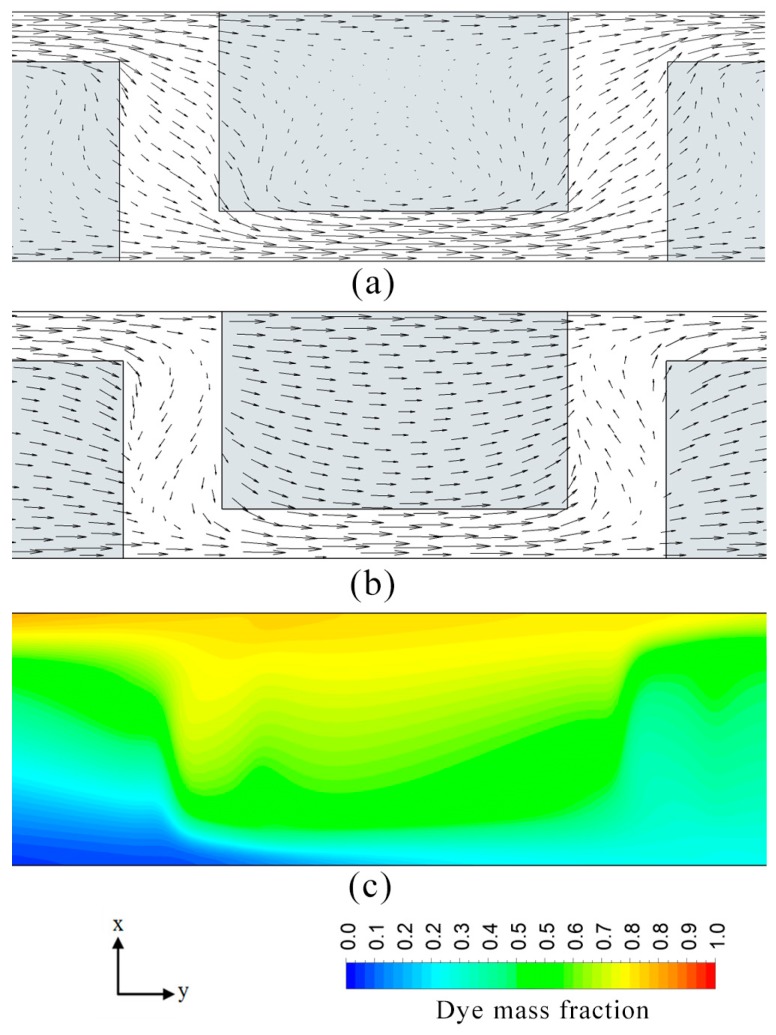
Flow and mixing visualization in the proposed micromixer in the *x*-*y* plane at *E* = 400 V/cm: (**a**) velocity vectors in *x*-*y* plane at *z*/*H*_c_ = 0.3; (**b**) velocity vectors at *z*/*H*_c_ = 0.6; and (**c**) Dye mass fraction distribution in *x*-*y y*-*x* at *z*/*H*_c_ = 0.5.

To further improve the mixing performance, a modification of the micromixer design by introducing space (*P*_S_) between the two adjacent heterogeneous patches was proposed and analyzed in this work. The proposed surface pattern is illustrated in Figure 15. The flow field and dye mass concentration distribution obtained from the simulation for the new design are shown in Figure 16. This spacing introduces an additional localized flow in a lateral direction, which enhances mixing between the two fluid streams. The velocity vector plots in Figure 16a,b show the localized lateral flows induced in the space between the two consecutive heterogeneous patches at *z*/*H*_c_ = 0.3 and 0.6, respectively. The additionally introduced spaces affect the shape of the induced wave. The wave form exhibited resemblance to a square wave as shown in Figure 16c, which enlarges the interfacial area between fluids.

It was found in this study that channel width and patch length have the most profound effect on mixing performance. An increase in the patch length and a decrease in the channel width enhanced the mixing performance significantly, however, it caused a critical reduction in the mass flow rate. The patch width was found to have a relatively small effect on the mixing performance. Similarly, the optimal channel height was found to be 10 μm. Based on these findings, a modified micromixer design was contrived. The following dimensions were selected for the modified micromixer design: *W*_c_/*H*_c_ = 12.5, *P*_L_/*W*_c_ = 1.06, *P*_w_/*W*_c_ = 0.70, *S* = 0.375, and *H*_c_ = 12.0 μm. In order to ensure sufficient mass flow rate, the values selected for the channel width and height were slightly greater than the optimal values. As discussed earlier, *P*_w_/*W*_c_ did not affect the mixing index in a significant manner in the reference geometry. However, in the case of *P*_L_/*W*_c_ = 1.06, a higher value of *P*_w_/*W*_c_ showed better mixing performance as it was observed to push the interfacial area between fluids away from the centerline towards the channel wall, thereby decreasing the diffusion distance.

**Figure 17:**
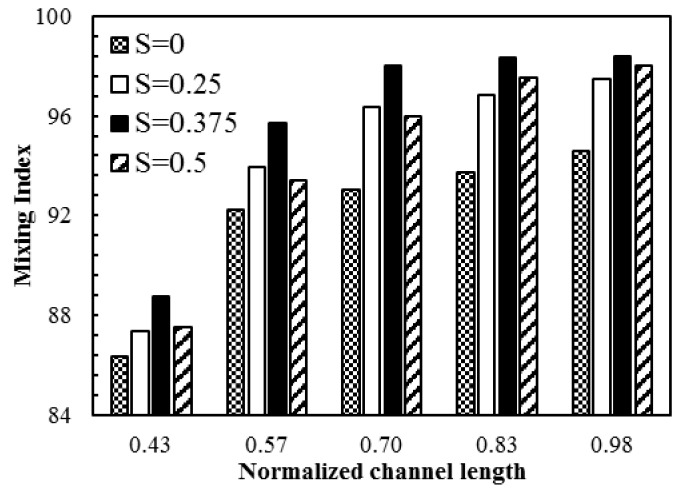
Comparison of the mixing index developments along the normalized channel length among the micromixers with different patch spaces at *E* = 400 V/cm.

**Figure 18:**
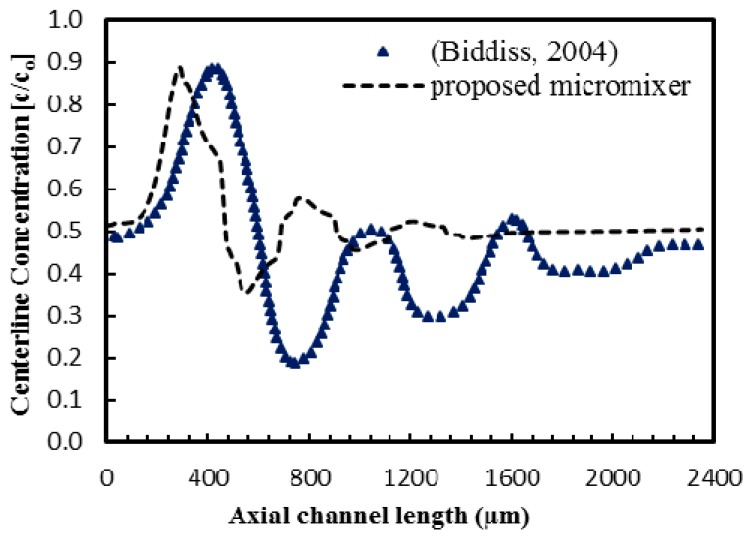
Comparison of normalized concentration distributions at centerline along the channel length between the proposed micromixer and the micromixer reported by Biddiss [8] at *E* = 280 V/cm.

The modified micromixer was then tested for different values of patch spacing to identify the optimum space value. Figure 17 shows the developments of mixing index along the channel length for different values of *S* (= *P*_S_/*P*_L_). All the modified designs with non-zero S show better mixing indices than the conventional design (*S* = 0). And, an optimum space (*S* = 0.375) shows the best mixing performance throughout the channel length. This optimum space (*S* = 0.375) was incorporated into the modified design. The new design shows excellent mixing performance, yielding a mixing index of 0.95 within less than a half of the channel length (1 mm) and a mixing index of 0.99 at the channel exit (2.4 mm) for applied electric field (**E**) of 280 V/cm. A comparative study was carried out between the modified design and the original design reported by Biddiss [8] for the micromixer with staggered heterogeneous surface charge pattern. The dimensions of the proposed and the Biddiss’ micromixers are compared in Table 2. The efficient heterogeneous patch configuration of the proposed design induces flow with greater interfacial area which enables rapid mixing over a shorter period of time compared to the Biddiss’ micromixer. Developments of centerline concentration along the channel length in the two micromixers are presented in Figure 18, where the proposed design shows rapid homogenization and outperforms the Biddiss’ design by achieving the same level of mixing with a shorter mixing section.

## 7. Conclusions

The present work performed a parametric performance analysis of an electroosmotic micromixer with staggered heterogenously charged patches was performed, and it proposed an improved micromixer design. Numerical analysis of flow and mixing was carried out using three-dimensional Poisson-Boltzman and Navier-Stokes equations with a diffusion-convection model for species concentration. The present numerical results for the species concentration profile across the channel width near channel exit were validated using experimental data with an average relative error of 0.59%. Effects of geometric and operating parameters on the flow structure and mixing were assessed in the parametric study. The numerical results suggested that, for *z*/*H*_c_ larger than 0.33, the flow vortices induced by the patches were critically weakened, and thus the flow above this height did not contribute significantly to the mixing. As the normalized voltage (*V*/ζ) decreased below 28.5, the mixing index increased rapidly and this voltage was selected as the reference value in the parametric study. The mixing index at the exit of the micromixer showed minimum value at the aspect ratio, *W*_c_/*H*_c_ = 6.65. Increasing the ratio of patch length to channel width (*P*_L_/*W*_c_) increased the mixing index monotonically, resulting in enhancement of the mixing index by 28% in a range of *P*_L_/*W*_c_ from 0.3 to 0.6. The mixing performance reached maximum at the ratio of patch length to channel width, *P*_w_/*W*_c_ = 0.3, but was not affected significantly by this parameter. Based on the analysis of the flow field, an improved design of the micromixer introducing a space (*P*_S_) between two adjacent heterogeneous patches was proposed in this work. The modified design induced an additional localized flow in the space between the two consecutive heterogeneous patches and changed the shape of induced flow wave, which enhanced mixing of fluids. The final design of the proposed micromixer was determined based on the results of the parametric study: *W*_c_/*H*_c_ = 12.5, *P*_L_/*W*_c_ = 1.06, *P*_w_/*W*_c_ = 0.70, *P*_S_/*P*_L_ = 0.375, and *H*_c_ = 12.0 μm. The modified micromixer design exhibited excellent mixing performance: a mixing index of 0.95 at less than a half of the channel length (1 mm) and 0.99 at the channel exit (2.4 mm) for applied electric field of 280 V/cm.

## Figures and Tables

**Table 1:** Reference values and ranges of the parameters.

No.	Parameter	Reference Value	Range
1	*V*/ζ	28.5	7.5–42.5
2	*W*_c_/*H*_c_	5.0	1.0–10
3	*P*_w_/*W*_c_	0.3	0.1–0.6
4	*P*_L_/*W*_c_	0.3	0.1–0.6

**Table 2:** Dimensions of the micromixer designs.

No.	Parameter	Proposed Design	Biddiss’ Design
1	*W*_c_/*H*_c_	12.5	25
2	*P*_L_/*W*_c_	1.06	1.5
3	*P*_w_/*W*_c_	0.7	0.45
4	*H*_c_	12 μm	8 μm
5	*S* (*P*_S_/*P*_L_)	0.375	0

## Nomenclature

A	area
$c_{i}$	mass fraction at sampling point *i*
$c_{m}$	optimal mass fraction
*d*	hydraulic diameter
*D*	diffusivity coefficient
E	electric field vector
$H_{c}$	microchannel Height
$k_{b}$	boltzmann constant
$L_{c}$	axial Length of microchannel
MI	mixing index
*n*	ionic number concentration
$P_{L}$	length of Heterogeneous Patch
$P_{w}$	width of Heterogeneous Patch
$P_{S}$	distance between two adjacent Heterogeneous Patches
Re	Reynolds number
*S*	normalized patch spacing
*u*	average velocity at channel exit
*V*	volume of fluid
$\vec{V}$	velocity vector
$W_{c}$	width of microchannel
$W_{i}$	width of microchannel
*x*, *y*, *z*	orthogonal coordinate system
*z_b_*	number of valence
ε	permittivity of fluid
$\mu_{f}$	dynamic viscosity
$\rho_{f}$	density of fluid
$\rho_{e}$	electric charge density
ζ	zeta potential
ψ	electric potential due to charge distribution within Debye layer
σ	standard variance
**Subscript**	
∞, b	bulk value
f	working fluid
